# Treatment uptake among notified cases of hepatitis C virus infection in Norway, 1990 to 2022: a registry-based study to monitor progress towards elimination

**DOI:** 10.2807/1560-7917.ES.2024.29.46.2400180

**Published:** 2024-11-14

**Authors:** Robert Whittaker, Håvard Midgard, Olav Dalgard, Hilde Kløvstad

**Affiliations:** 1 Department of Infection Control and Vaccines, Norwegian Institute of Public Health, Oslo, Norway; 2 Department of Gastroenterology, Oslo University Hospital, Oslo, Norway; 3 Department of Infectious Diseases, Akershus University Hospital, Lørenskog, Norway; 4 Institute of Clinical Medicine, University of Oslo, Oslo, Norway

**Keywords:** Hepatitis C, surveillance, elimination, Norway, electronic health registry

## Abstract

**Background:**

Hepatitis C virus (HCV) infection is targeted for elimination as a public health threat by 2030. The World Health Organization goal for treatment uptake is ≥ 80% among cases diagnosed with an active HCV infection (RNA- or core antigen-positive), maintained for 2 years.

**Aim:**

To calculate treatment uptake, time from diagnosis to treatment start and complete treatment dispensation among notified cases of HCV infection in Norway.

**Method:**

We linked national data on notified cases diagnosed from 1990–2022 to dispensed prescriptions for HCV treatment from January 2004–February 2023 and data on residence status at the end of 2022. We described treatment uptake by demographic and epidemiological characteristics. We calculated the median number of months from diagnosis to treatment start by year. For direct-acting antiviral treatment periods, complete dispensation was based on the treatment and number of defined daily doses.

**Results:**

Among 12,178 notified cases that had an active infection at diagnosis and were still resident, 10,091 (83%) had received treatment. Uptake among resident cases was > 80% in 2021 and 2022. The median number of months from diagnosis to treatment decreased over time to 3 (interquartile range: 1–5) among cases diagnosed in 2021 and 2022. From 2015–22, 89–93% of direct-acting antiviral treatment periods annually had complete treatment dispensed.

**Conclusion:**

Norway has achieved the elimination goal for treatment uptake among notified cases diagnosed with an active HCV infection. This highlights the benefit of a strategy that includes unrestricted, simplified and integrated treatment options.

**Key public health message**

**What did you want to address in this study?**

Hepatitis C virus (HCV) infection is a largely chronic but treatable infection that is targeted for elimination as a public health threat by the World Health Organization. One of the elimination goals is that at least 80% of people with an active HCV infection receive treatment. We aimed to determine progress towards this goal in Norway, a country with an epidemic of HCV infection predominantly driven by injecting drugs.

**What have we learnt from this study?**

In both 2021 and 2022, treatment uptake among infected individuals who still lived in Norway at the end of the year was 80% or higher. The time from diagnosis to treatment decreased from several years in the early 2000s to around 3 months in 2021 and 2022. From 2015–22, around 90% of those treated annually had received a complete treatment course.

**What are the implications of your findings for public health?**

Norway is one of the first countries globally to document achievement of the elimination goal for treatment uptake. This highlights the benefit of offering treatment to all diagnosed with HCV infection, and having simplified and integrated treatment options available. Initiatives to further increase uptake and maintain elimination in Norway should be considered.

## Introduction

Hepatitis C virus (HCV) is blood-borne and causes an often chronic, asymptomatic infection that, if untreated, may lead to progressive liver disease and death [1]. In 2022, there were an estimated one million new infections and 240,000 HCV-related deaths globally [2]. No vaccine against HCV infection is available. Until 2010, HCV infection was treated with interferon and ribavirin, with treatment courses lasting up to 72 weeks accompanied by frequent and often serious side effects and cure rates averaging around 50% [3]. The introduction of protease inhibitors in 2011, combined with interferon and ribavirin, provided some increase in effectiveness for patients with genotype 1 infection, but not tolerability [3]. Since 2014, interferon-free direct-acting antiviral (DAA) treatment has been available. Interferon-free DAAs are taken orally, have few side effects, are highly effective (≥ 95% cure rate), have courses of short duration (8–12 weeks) and are effective against all genotypes (1–6) [3]. The elimination of HCV infection as a public health threat has therefore become a feasible global goal [2].

The World Health Organization (WHO) aims to achieve elimination by 2030 [2]. WHO defines elimination by disease impact and healthcare coverage targets. The goal for treatment uptake is ≥ 80% in cases diagnosed with an active infection (i.e. a positive HCV RNA or core antigen test) initiating treatment, maintained for at least 2 years [4]. In 2022, WHO estimated global treatment uptake to be 20% [2]. Currently, only 12 countries globally (of which seven are countries of the European Union/European Economic Area (EU/EEA)) are reported to be on track to achieving elimination by 2030 [5], including Norway (population 5.4 million).

As in many European countries, the epidemic of HCV infection in Norway has been concentrated among people who inject drugs (PWID) [6,7]. Immigrants from high prevalence countries are the second most important risk group for chronic infection [6]. During the ‘interferon’ era, treatment involved complicated specialist care [8]. Once interferon-free DAAs were introduced, treatment models could be simplified. Initially, DAA treatment was costly and only offered to those with notable liver fibrosis [9-11]. However, a dramatic reduction in price allowed unrestricted DAA treatment in Norway from February 2018 [12,13]. This policy is recommended by the WHO [4] and in place in most EU/EEA countries [14].

The main care pathway for HCV infection in Norway is diagnosis by the primary care physician and referral to specialist care. However, this model is of limited value for PWID, who are at high risk of loss to follow-up [15]. In line with WHO recommendations, decentralised models of care have been developed [4]. Several clinics for opioid agonist therapy (OAT) and some low-threshold outreach municipality services have implemented integrated test-and-treat services [16,17]. Testing and treatment are also available in prison. Furthermore, some low-threshold outreach services for PWID have been established in both large cities and small towns, where nurses offer point-of-care HCV RNA testing, assessment for liver fibrosis and prescription of treatment (via phone call to specialist) in one visit [18,19]. Treatment uptake among people who had recently injected drugs has consequently increased markedly, compared with the interferon era [20]. It is also of importance that the campaign to eliminate HCV infection in Norway has been conducted in a setting with high uptake to OAT and widespread access to sterile needles and syringes [6].

Treatment uptake for HCV infection in Norway has not been studied beyond 2018 [6]. Data on treatment uptake are also lacking internationally [21,22]. Such data are essential to monitor progress towards elimination and inform strategies for achieving and maintaining this goal. We linked national electronic health registry data to calculate treatment uptake, time from diagnosis to treatment start and the proportion dispensed a complete course among notified cases of HCV infection in Norway until the end of 2022. We also explored under-reporting of diagnosed cases.

## Methods

### Data sources

#### Norwegian Surveillance System for Communicable Diseases

The Norwegian Surveillance System for Communicable Diseases (MSIS) has maintained a national database on mandatorily notifiable infectious diseases in Norway since 1975, compiling cases reported by clinicians and laboratories. Cases of HCV infection (both primary and reinfection) have been reported with personally identifiable data since 1990, following the discovery of HCV in 1989 [1]. From 1990–91 and 2008–15, all anti-HCV antibody, HCV RNA and HCV core antigen positive cases were notifiable. From 1992–2007, only acute cases (i.e. known recent infections) were notifiable. Chronic cases diagnosed in this period were notified from 2008 onwards. Since 2016, only cases with a positive RNA or core antigen test, i.e. an active infection, have been notifiable. Acute and chronic cases are not differentiated [23].

#### Norwegian Prescribed Drug Registry

The Norwegian Prescribed Drug Registry (LMR) contains personally identifiable data on medicines dispensed by prescription from pharmacies in Norway since 2004 [24]. Specific medicines are identified using anatomical therapeutic chemical (ATC) codes [25].

#### National Population Register

The National Population Register (FR) contains information on everyone who resides or has resided in Norway [26].

### Data linkage and dataset

Using a deidentified project specific number based on the Norwegian national identity number (‘Fødselsnummer’) or D number for temporary residents (the allocation of these numbers is described in [26]), we linked notified cases of HCV infection registered in MSIS with a date of diagnosis from January 1990–December 2022 to: (i) dispensed prescriptions in LMR from January 2004–February 2023 with ATC codes for medicines for treating HCV infection (antivirals for the treatment of HCV infections (code J05AP) and interferon-alpha 2a/2b (codes L03AB04, -05, -10 and -11)); and (ii) registrations in FR, with data updated until the end of December 2022.

All data endpoints reflect the latest available data at the time of extraction. Data from MSIS included year of birth, month and year of diagnosis, sex, country of birth, county of residence, test method, reported route of transmission and reported place of infection. Data from LMR included month and year of each dispensed prescription, ATC code (at the 5th level), the number of packs and defined daily doses (DDD) dispensed and county of residence at prescription. Data from FR included current residence status (e.g. resident, died, out-migrated), year of residence status (e.g. 2022 for all those still resident, year of death for those who had died), and current or most recent county of residence.

The MSIS is a passive surveillance system, meaning reporting is not prompted. To ensure as complete a dataset as possible on treatment for HCV infection and to investigate potential under-reporting to MSIS, we also received LMR data on dispensed prescriptions of medicines for HCV treatment for individuals who could not be linked to a notified case of HCV infection in MSIS.

### Data analysis

#### Aggregation of treatment periods

The length of treatment for HCV infection has ranged from 8–72 weeks [8,13]. Individuals infected with HCV may also undergo several treatment periods, either because of incomplete treatment, treatment failure or reinfection. Therefore, we aggregated prescriptions into treatment periods per person, based on the month of the prescription and medicine dispensed. In the Supplement, part 1, we detail this aggregation and describe treatment periods from 2004–22 by year, number of periods, number of individuals, medicine dispensed and whether the treatment period was able to be linked to a case in MSIS.

#### Treatment uptake among notified cases

We defined treatment uptake among notified cases as having ever been dispensed treatment for HCV infection. For individuals registered more than once in MSIS, i.e. reinfections, we only considered treatment periods to be related to the case if they occurred in the time period before diagnosis of a subsequent infection. Cases were considered to be untreated if they had received treatment, but were diagnosed with an active infection after the month of their last dispensed prescription, or only ever received ribavirin, which has limited efficacy against HCV infection and not part of Norwegian clinical guidelines [3,8].

#### Time from diagnosis to treatment start

For cases diagnosed from 2004, the first year with available treatment data, we also described the median time and interquartile range (IQR) from diagnosis to the first treatment period that occurred in or after the month of diagnosis.

#### Complete direct-acting antiviral treatment course dispensed

For each treatment period that started by November 2022 (a minimum 3 months of follow-up) with the prescription of at least one DAA, we determined the dispensation of a complete treatment course, based on the DAA(s) and number of DDD prescribed in the period, according to Norwegian clinical guidelines [8-13] (Table 1).

**Table 1:** Number of defined daily doses to define being dispensed a complete treatment course with a direct-acting antiviral, by medicine

ATC code^a^	Name of medicine	Class of medicine	Number of DDD
J05AP02	Telaprevir	Protease inhibitor	84
J05AP03	Boceprevir	Protease inhibitor	168
J05AP05	Simeprevir	Protease inhibitor	84
J05AP07	Daclatasvir	Polymerase inhibitor	84
J05AP08 + J05AP01 + L03AB10 or L03AB11	Sofosbuvir + ribavirin + interferon	Polymerase inhibitor + ribavirin + peg-interferon-alpha	84
J05AP08 + J05AP01	Sofosbuvir + ribavirin	Polymerase inhibitor + ribavirin	84^b^
J05AP08 + J05AP05	Sofosbuvir + simeprevir	Polymerase inhibitor + protease inhibitor	168^c^
J05AP09	Dasabuvir	Polymerase inhibitor	84
J05AP51	Sofosbuvir/ledipasvir	Fixed dose combination	56^b^
J05AP51 + J05AP01	Sofosbuvir/ledipasvir + ribavirin	Fixed dose combination + ribavirin	84
J05AP53	Ombitasvir/paritaprevir/ritonavir	Fixed dose combination	84
J05AP54	Elbasvir/grazoprevir	Fixed dose combination	84
J05AP55	Sofosbuvir/velpatasvir	Fixed dose combination	84^b^
J05AP56	Sofosbuvir/velpatasvir/voxilaprevir	Fixed dose combination	84
J05AP57	Glecaprevir/pibrentasvir	Fixed dose combination	56

ATC: anatomical therapeutic chemical; DDD: defined daily dose.

^a^ Different combinations of ATC codes are only presented and analysed separately if they changed the number of DDD required for complete treatment dispensation than an individual DAA alone (e.g. J05AP51 vs J05AP51 + J05AP01).

^b^ The course length for some treatments could vary, for example by genotype and stage of liver disease [8-13], but data were lacking on these parameters. In the Supplement, part 2, we present results where we consider an alternative definition for complete treatment dispensation and compare to results using the values in Table 1.

^c^ 84 for each ATC code.

#### Individuals treated for HCV infection not notified to MSIS

Individuals treated for HCV infection are diagnosed before treatment start and should also be tested during treatment follow-up. To explore potential under-reporting to MSIS, we identified individuals who had received treatment for HCV infection and had a linkable identity number registered in LMR, but were not able to be linked to a notified case of HCV infection in MSIS.

For each outcome, we described cases by year of diagnosis, year of treatment, treatment received, age, sex, region of residence, country of birth, reported route of transmission, reported place of infection, test method and/or current residence status. All analyses were conducted in Stata version 18 (Stata Corporation).

## Results

### Treatment uptake among notified cases

Among 23,484 notified cases of HCV infection registered in MSIS until the end of 2022, 22,048 (94%) could be linked to LMR and FR. There were 21,987 (99%) unique individuals and 61 (0.3%) were registered twice (i.e. reinfection). Of these 21,987 individuals, 16,955 (77%) were still resident in Norway at the end of 2022, 4,404 (20%) had died, 539 (2.5%) had out-migrated, 24 (0.1%) had other (for example ‘disappeared’) residence status and 65 (0.3%) could not be linked to FR. The median age at diagnosis was 38 years (IQR: 30–48) and most cases were male (66%), born in Norway (81%) and reported to be infected in Norway by injecting drugs (86%). Detailed characteristics and treatment uptake among notified cases are presented in Table 2.

**Table 2:** Characteristics of notified cases of hepatitis C virus infection and those with treatment dispensed, according to test method and residence status, Norway, 1990–2022 (n = 22,048)

Characteristics	All notified cases			Test method RNA or antigen detection			Still resident at the end of 2022			Test method RNA or antigen detection and still resident at the end of 2022		
	Number of cases	Dispensed treatment		Number of cases	Dispensed treatment		Number of cases	Dispensed treatment		Number of cases	Dispensed treatment	
		n	%		n	%		n	%		n	%
Overall	22,048	13,186	60	15,100	11,285	75	17,013	11,767	69	12,178	10,090	83
Year of diagnosis^a^												
1990–1991	1,608	428	27	345	270	78	741	360	49	248	229	92
1992–2007	2,792	1,499	54	1,604	1,132	71	2,001	1,290	64	1,183	975	82
2008–2010	6,936	3,987	57	4,669	3,339	72	5,251	3,496	67	3,542	2,928	83
2011–2013	4,121	2,436	59	2,634	1,948	74	3,326	2,188	66	2,128	1,744	82
2014–2015	2,260	1,381	61	1,518	1,142	75	1,879	1,253	67	1,263	1,035	82
2016–2017	1,660	1,347	81	1,660	1,347	81	1,416	1,218	86	1,416	1,218	86
2018–2019	1,376	1,153	84	1,375	1,152	84	1,227	1,065	87	1,226	1,064	87
2020–2021	838	667	80	838	667	80	756	630	83	756	630	83
2022^b^	457	288	63	457	288	63	416	267	64	416	267	64
Age in years at diagnosis^c^												
0–2^d^	46	16	35	31	14	45	44	16	36	29	14	48
3–14	59	41	69	43	37	86	54	41	76	41	37	90
15–24	2,230	1,267	57	1,386	1,017	73	1,861	1,202	65	1,240	971	78
25–34	6,262	3,601	58	3,944	2,964	75	4,951	3,317	67	3,335	2,733	82
35–44	5,958	3,556	60	4,071	3,065	75	4,706	3,206	68	3,336	2,768	83
45–54	4,696	3,006	64	3,425	2,638	77	3,582	2,604	73	2,684	2,295	86
55–64	2,190	1,412	64	1,758	1,289	73	1,511	1,159	77	1,248	1,068	86
≥ 65	607	287	47	442	261	59	304	222	73	265	204	77
Sex												
Female	7,554	4,404	58	5,033	3,782	75	6,301	4,068	65	4,333	3,490	81
Male	14,494	8,782	61	10,067	7,503	75	10,712	7,699	72	7,845	6,600	84
Region of residence at diagnosis^e^												
Mid Norway	1,958	1,237	63	1495	1,126	75	1,563	1,114	71	1236	1,018	82
North Norway	1,689	1,132	67	1358	1,041	77	1,367	1,006	74	1123	934	83
Oslo	4,269	2,266	53	2,465	1,855	75	2,963	1,966	66	1,866	1,603	86
South-East Norway (excluding Oslo)	9,815	6,032	61	7,199	5,418	75	7,747	5,414	70	5,872	4,877	83
West Norway	4,263	2,492	58	2,531	1,818	72	3,359	2,258	67	2,069	1,649	80
Unknown	54	27	50	52	27	52	14	9	64	12	9	75
Country of birth												
Norway	16,839	10,450	62	12,165	9,036	74	13,489	9,401	70	9,881	8,139	82
Europe (excluding Norway)^f^	1,996	1,337	67	1,549	1,193	77	1,546	1,164	75	1,218	1,039	85
Other^f^	1,859	1,197	64	1,294	999	77	1,446	1,038	72	1,016	867	85
Unknown^g^	1,354	202	15	92	57	62	532	164	31	63	45	71
Reported route of transmission												
Injecting drugs	10,729	6,777	63	7,986	5,963	75	8,707	6,137	70	6,589	5,403	82
Blood contact^h^	670	465	69	532	424	80	520	402	77	418	369	88
Heterosexual contact	79	63	80	67	56	84	73	60	82	63	53	84
Homosexual contact	51	39	76	47	37	79	48	38	79	44	36	82
Sexual contact, unspecified	433	281	65	307	242	79	356	253	71	255	217	85
Perinatal	70	45	64	61	42	69	67	45	67	58	42	72
Other	41	25	61	26	19	73	31	22	71	20	17	85
Unknown^i^	9,975	5,491	55	6,074	4,502	74	7,211	4,810	67	4,731	3,953	84
Reported place of infection												
Norway	16,115	9,753	61	11,229	8,273	74	12,755	8,731	68	9,025	7,418	82
Overseas	2,101	1,392	66	1,573	1,214	77	1,639	1,212	74	1,243	1,054	85
Unknown	3,832	2,041	53	2,298	1,798	78	2,619	1,824	70	1,910	1,618	85
Test method												
Antibody detection	5,693	1,727	30	NA			4,361	1,531	35	NA		
RNA/antigen detection^j^	15,100	11,285	75				12,178	10,090	83			
Unknown^g^	1,255	174	14				474	146	31			

NA: not applicable.

^a^ Aggregated into periods based on the timing of changes in the national case definition for cases of HCV infection, notable changes in available treatment or treatment guidelines and the COVID-19 pandemic. Further details are given in the Supplement, part 3. In total, 1,512 (6.9%) cases had a date of registration in MSIS, but not a date of diagnosis. Of these, 1,300 (86%) were registered in 1990–1991 and 183 (12%) in 1992–2007. All cases reported from 2015 onwards had a date of diagnosis.

^b^ Lower treatment uptake in 2022 is likely due to data availability at the time of extraction (February 2023).

^c^ The current age, up to the end of 2022, among those still resident but who had not received treatment is presented in the Supplement, part 3.

^d^ All cases aged 0–2 years had turned 3 years of age by the end of 2022 and were thus now eligible for treatment [13].

^e^ A more detailed breakdown by county (2023 classification) is presented in the Supplement, part 4. The current county of residence (2023 classification) among those still resident but who had not received treatment is also presented in the Supplement, part 3.

^f^ A more detailed breakdown of the groups ‘Europe (excluding Norway)’ and ‘Other’ is presented in the Supplement, part 4.

^g^ Almost exclusively reported in 1990–91 (for all cases, country of birth: n = 1,068, 79%; test method: n = 1,110, 88%).

^h^ Such as a needle-stick injury or blood transfusion.

^i^ Of these, 9,975 cases, 6,304 (63%) were born in Norway, and thus it is most likely that the vast majority of these 6,304 were infected by injecting drugs.

^j^ In total, 15 cases were reported with antigen detection as the test method.

Treatment for hepatitis C virus (HCV) infection defined as being dispensed a prescription for ATC codes J05AP and/or L03AB04, -05, -10 and -11. Cases who received treatment but were diagnosed with an active infection after the month of their last dispensed prescription (n = 79 among all cases) or only ever received ribavirin (limited efficacy against HCV infection and not part of Norwegian clinical guidelines [3,8]) (n = 4 among all cases) were considered untreated cases. Further details on the treatment courses dispensed by year are presented in the Supplement, part 1.

Of 13,186 treated cases, 10,670 (81%) had received a DAA. Among 3,746 cases first treated with ribavirin/interferon, 1,230 (33%) later received a DAA. This proportion was higher among those diagnosed with an active infection (1,092/2,824, 39%) than positive anti-HCV antibody test but unknown HCV RNA or antigen status (123/857, 14%) or unknown test method (15/50, 30%). Specific details on treatment type are presented in the Supplement, part 1.

Among cases who first received treatment in 2021 or 2022 (n = 1,246), 114 (9.1%) were diagnosed between 2016–2019, and 463 (37%, of which 62 with anti-HCV antibody detection as test method) were diagnosed between 1990–2015. A more detailed breakdown of year of treatment by year of diagnosis is presented in the Supplement, part 5.

**Figure 1:**
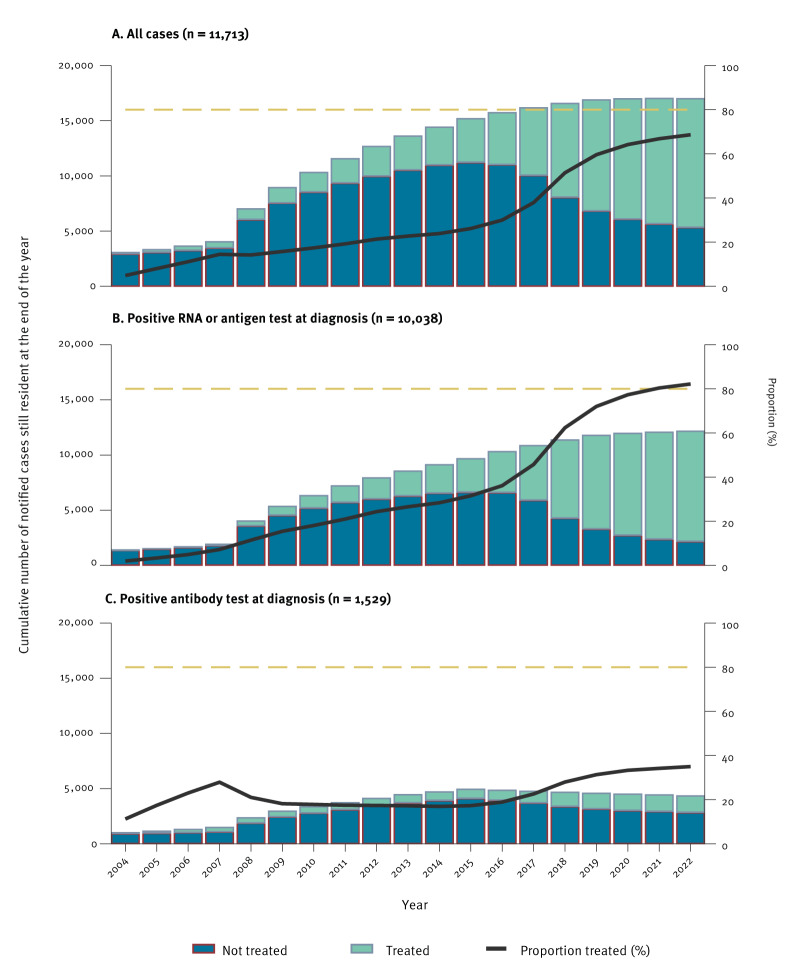
Cumulative number of treated and untreated notified cases of hepatitis C virus infection that were still resident at the end of each year and proportion treated, by year and test method, Norway, 2004–2022

Among cases diagnosed with an active infection and still resident, treatment uptake at the end of each year increased from 28% in 2014 (the first year when interferon-free DAA became available) to 77% in 2020, 80% in 2021 and 82% in 2022 (Figure 1B). Among those still resident at the end of 2022, treatment uptake was over or near 80% in most characteristics presented in Table 2. Treatment uptake was < 75% among cases diagnosed in 2022, aged 0–2 years at diagnosis or reported to have been infected perinatally (Table 2). In 2022, 173 cases were diagnosed from September 2022 onwards, 164 (95%) still resident. Excluding these 164, treatment uptake in 2022 among those still resident increased from 64% (267/416) to 71% (179/252). Among cases diagnosed in the first 8 months of the year in 2020 and 2021, treatment uptake by February the following year was 70% (182/259) and 65% (195/299), respectively. Among the 58 infected by perinatal transmission, 25 (43%) were aged 0–2 years at diagnosis. Excluding these cases, treatment uptake among those infected perinatally was 88% (29/33). 

The dashed yellow line on 80% reflects the World Health Organization elimination goal for treatment uptake. Cases who were still resident at the end of 2022 are considered to have been consistently resident from the year of diagnosis. Other cases leave the cohort in the year they were registered as no longer being resident (died, out-migrated, etc.). Further details and the data behind the figure are presented in the Supplement, part 6. Additional details on the treatment courses dispensed by year are presented in the Supplement, part 1.

Treatment uptake in 2022 was 35% (1,531/4,361) among resident cases that were anti-HCV antibody positive with unknown HCV RNA or antigen status and 31% (146/474) among cases with unknown test method (Table 2, Figure 1).

Among all 17,013 notified cases that were still resident, 5,246 (31%) were untreated; 2,088 (40%) diagnosed by RNA or antigen detection, 2,830 (54%) by anti-HCV antibody detection with unknown RNA or antigen status and 328 (6.3%) with unknown test method. Over half the untreated cases were aged ≥ 45 years (n = 3,516, 67%) at the end of 2022. Among cases diagnosed by RNA or antigen detection, 1,210 (58%) were aged ≥ 45 years. Additional characteristics of untreated cases are presented in the Supplement, part 3.

### Time from diagnosis to treatment start

**Figure 2:**
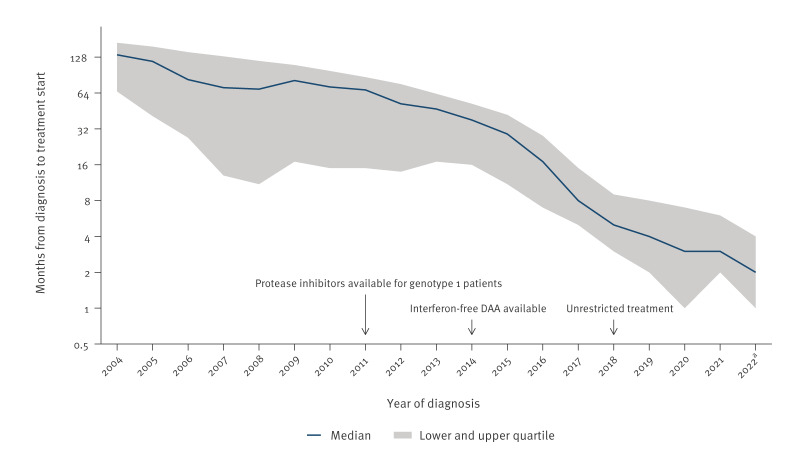
Median number of months from diagnosis to treatment start, notified cases of hepatitis C virus infection who had received treatment, Norway, 2004–2022 (n = 11,883)

The time from diagnosis to treatment start decreased over time, reaching a median of 5 months (IQR: 3–9) among cases diagnosed in 2018 (n = 631), when all treatment restrictions were removed. For cases diagnosed in 2021 or 2022 (n = 580), the median was 3 months (IQR: 1–5) (Figure 2).

DAA: direct-acting antiviral.

^a^ Given that we did not have access to treatment data beyond the end of February 2023, data for 2022 are preliminary.

The y-axis is a logarithmic scale. The data behind the figure are presented in the Supplement, part 7. Further details on the treatment courses dispensed by year are presented in the Supplement, part 1.

### Complete direct-acting antiviral treatment course dispensed

Among 10,533 notified cases who had started a DAA treatment period by November 2022, 9,893 (94%) had ever had at least one complete course dispensed. At least 90% of cases in all characteristics presented in Table 2 had had at least one complete course dispensed (data on file), including 8,982 of 9,524 (94%) cases still resident at the end of 2022.

The 10,533 cases had 11,661 different DAA treatment periods, of which 10,494 (90%) had a complete course dispensed. This proportion increased from 53% in 2011, when protease inhibitors were the only DAA prescribed, to 84–93% each year from 2014, when interferon-free DAA were available (data by year are presented in the Supplement, part 2).

### Individuals treated for HCV infection not registered in MSIS

Overall, 1,175 individuals (8.2% of 14,313 total treated individuals, as described in the Supplement, part 1) with a linkable identity number registered in LMR had been dispensed treatment for HCV infection, but not been registered in MSIS. All had received at least one treatment period from 2008 onwards, from which time all active infections have been notifiable. From 2008–22, 17,648 cases with a linkable identify number were notified to MSIS (Table 2). Including these 1,175, this suggests under-reporting of at least 6.2% (1,175/18,823) in this period. From 2020–2022, among 2,853 individuals who received at least one treatment period, 219 (7.7%) were not registered in MSIS. Considering 1,295 notified cases with a linkable identify number (Table 2), this suggests under-reporting of at least 14% (n = 219/1,514) in this period. Among 1,293 different DAA treatment periods which started by November 2022 among individuals not registered in MSIS, 1,148 (89%) had a complete course dispensed.

## Discussion

In Norway, treatment uptake among notified cases diagnosed with an active HCV infection was above 80% in 2021 and 2022. This indicates that Norway has achieved the WHO global elimination target for treatment of HCV infection [4], alongside countries like Iceland and Egypt [22]. Most notified cases in Norway now start treatment within a few of months of diagnosis. Also, cases treated in recent years included a notable proportion that were first diagnosed several years prior, indicating that cases diagnosed before restrictions on treatment access were removed continue to come for treatment. While WHO guidelines specify ‘treatment initiation … with DAAs’ [4], we do not disregard treatment with interferon/ribavirin from our calculation. A study among three Norwegian hospitals found a success rate of interferon/ribavirin treatment around 70% [27]. In our study, 40% of cases diagnosed with an active infection and initially treated with ribavirin/interferon later received a DAA. This suggests a high rate of DAA retreatment among those initially unsuccessfully treated with ribavirin/interferon.

Although not necessary for the validation of elimination [4], measures of treatment success remain important to best monitor treatment strategies. Since the introduction of interferon-free DAA, around 90% of treatment periods in Norway have had a complete course dispensed. High treatment completion likely reflects high cure rates [3,28]. Sustained virological response may also be attained despite suboptimal adherence [29].

High treatment uptake in Norway reflects the benefits of the strategy and measures implemented thus far in a setting with an epidemic predominantly driven by injecting drug use. The impact on the disease burden has been demonstrated. The prevalence of HCV infection among PWID has declined from around 50% in 2017 to under 10% in 2022 [6,30]. Modelled incidence of new infections among PWID and observed incidence of HCV-related deaths are below WHO impact targets [6,30]. However, treatment uptake in Norway appears to have stagnated in recent years and over 10% of cases diagnosed annually since all treatment restrictions were removed have not been treated. Also, a quarter of cases diagnosed and treated in recent years took 6 months or more to start treatment. While we do not know the extent to which the COVID-19 pandemic may have influenced this finding, this is nonetheless a timely juncture to reflect on where improvements could be made. New diagnoses and treatment need are unlikely to curtail very soon, with an estimated 3,000 chronic infections among current and former PWID and immigrants at the end of 2022 [30].

Lower uptake among cases aged < 3 years at diagnosis likely reflects treatment eligibility criteria [13]. Lower uptake in 2022 is likely due to data availability at the time of extraction. While our study provides valuable background data, further exploration beyond a descriptive analysis was hampered by the limited range of characteristics available, low data completeness for some of these and a lack of data on a range of important confounders, such as recent drug use [19,31], use of services where engagement in treatment is more likely, like OAT [20,32], and contraindications to treatment, such as pregnancy or breastfeeding [13].

Other studies provide further insight into where additional gains can be made. Studies among PWID in Oslo, Norway’s capital and largest city, have pointed to higher uptake among men and those in OAT [20,33]. This is supported by similar findings in other studies [32,34], highlighting the potential benefit of further increasing uptake in OAT and more targeted treatment models for female PWID. There is also ever-growing evidence from a range of services, including OAT, prisons, needle and syringe programmes, and in-patient hospital wards [16,35,36] supporting the WHO recommendation of same-site test-and-treat models [4]. On-site point-of-care testing itself may also increase treatment uptake and reduce the time from diagnosis to treatment [37]. This highlights further potential for the decentralisation and integration of services in Norway, where test-and-treat models are restricted to some OAT clinics and low-threshold outreach services for PWID [16,18,19].

Furthermore, in our study over 5,000 cases (of which around 2,000 diagnosed with an active infection) were still resident and untreated at the end of 2022. Including all resident cases, regardless of test method, overall treatment uptake dropped to 69%, although this does not challenge our conclusion of achieving the elimination goal, as defined by WHO [4]. Also, one may expect a notable proportion of these untreated cases to have spontaneously cleared their acute infection, especially given that the majority were diagnosed by anti-HCV antibody only and that all treated cases, i.e. a cohort of cases who did develop a chronic infection, are excluded. However, the treatment need of these untreated cases is unknown. Over 3,500 cases (or 1,200 among those diagnosed with an active infection) are now at an age that places them at increasing risk of cirrhosis [1]. The ‘retrieval’ [38] and ‘call in’ [39] initiatives in the Netherlands and Denmark provide inspiration as to how cases lost to follow-up can be reached. The registry data in this study could underpin a similar initiative in Norway.

This study includes 94% of cases of HCV infection ever notified in Norway and all dispensed treatment prescriptions for HCV infection since 2004. However, there are some limitations with the secondary use of registry data that need to be acknowledged. Some limitations with the dataset regarding confounding and data completeness have been mentioned above. Further limitations include that we only had data on dispensed prescriptions, not whether treatment was prescribed but not dispensed, details on adherence or whether sustained virological response was achieved. This underscores the added value of supplementary clinical studies in the surveillance of the elimination goals for HCV infection, like the European Centre for Disease Prevention and Control sentinel surveillance project on treatment outcomes [40]. Furthermore, as a passive surveillance system and with a case definition that has varied over time, MSIS is prone to under-reporting of diagnosed cases. A higher proportion of unnotified treated individuals in recent years may reflect cases first diagnosed several years ago that are now receiving treatment, where the clinician is unaware that the case has not been notified. Also, multiple treatment periods for the same case may reflect unnotified reinfections. However, as all these individuals had been treated, we do not have any reason to believe that this unduly affects our conclusions. The gradual establishment of the national microbiology database in Norway from 2020, containing case-based data on tests conducted by all medical laboratories [41], will facilitate a transition towards more automated case finding, minimising under-reporting and improving HCV reinfection surveillance in Norway. From 2024, all medical laboratories were electronically and automatically reporting all test results for hepatitis C to the national microbiology database.

## Conclusion

We find that Norway has achieved the WHO elimination goal for treatment uptake among notified cases diagnosed with an active HCV infection. This highlights the benefit of a strategy that includes unrestricted, simplified and integrated treatment options, and low-threshold and outreach services. Potential remains to improve treatment uptake, and further initiatives should be considered in efforts to maintain achievement of this elimination goal.

## Supplementary Data

753000 Supplement
