# Supplementary material for: Treatment uptake among notified cases of hepatitis C virus infection in Norway, 1990 to 2022: a registry-based study to monitor progress towards elimination
Source: Euro Surveill. 2024 Nov 14;29(46):2400180. doi: 10.2807/1560-7917.ES.2024.29.46.2400180 (PMC11565653; doi:10.2807/1560-7917.ES.2024.29.46.2400180)
Supplement: Supplement [file 24-00180_WHITTAKER_Supplement.pdf]

Treatment uptake among notified cases of hepatitis C virus infection in Norway, 1990 to 2022: a registry-based study to monitor progress towards elimination.

Supplement

This supplementary material is hosted by Eurosurveillance as supporting information alongside the article "Treatment uptake among notified cases of hepatitis C virus infection in Norway, 1990 to 2022: a registry-based study to monitor progress towards elimination", on behalf of the authors, who remain responsible for the accuracy and appropriateness of the content. The same standards for ethics, copyright, attributions and permissions as for the article apply. Supplements are not edited by Eurosurveillance and the journal is not responsible for the maintenance of any links or email addresses provided therein.

Contents

1. Treatment periods for hepatitis C virus (HCV) infection in Norway, 2004 – 2022 2
2. Results from alternative definition of complete DAA treatment course dispensed 6
3. Characteristics of notified cases of HCV infection who were untreated and still resident at the end of 2022 8
4. Treatment uptake by county of residence and country of birth 11
5. Year of treatment by year of diagnosis 13
6. Data behind figure 1 14
7. Data behind figure 2 16
8. References 17

1. Treatment periods for hepatitis C virus (HCV) infection in Norway, 2004 – 2022

Definition of treatment periods

The Norwegian Prescribed Drug Registry contains personally identifiable data on medicines dispensed by prescription from pharmacies in Norway since 2004 [1]. Specific medicines are identified using Anatomical Therapeutic Chemical (ATC) codes [2]. ATC codes for medicines for treating HCV infection include antivirals for the treatment of HCV infections (ATC codes J05AP) and interferon-α 2a/2b (ATC codes L03AB04, -05, -10 and -11).

The length of treatment for HCV infection has ranged from 8 – 72 weeks [3-8]. Persons infected with hepatitis C may also undergo several treatment periods, either due to treatment incompletion, treatment failure or reinfection. Therefore, we aggregated prescriptions into treatment periods per person, based on the month of the prescription and medicine dispensed. A treatment period included prescriptions where:

- There was ≤ 1 month between prescriptions, except when a new class of direct-acting antiviral (DAA) was dispensed for the first time (protease inhibitors (J05AP02 – J05AP05), polymerase inhibitors (J05AP07 – J05AP09) or fixed dose combinations (J05AP51 – J05AP57)).
- There were 2 – 3 months between prescriptions, but no change in the medicines dispensed.
- There were ≤ 6 months between prescriptions, when interferon and/or ribavirin (J05AP01) were the only medicines dispensed and prior to 2014. This is because the dosing and frequency of prescriptions for these medicines could vary, until 2014 from which time interferon and ribavirin were only recommended to be prescribed in 12-week courses to specific patients [4].
- Continued prescription of ribavirin and/or interferon, following a treatment period with a DAA, were included as part of the previous DAA treatment period.

Treatment courses by treatment period

Among 90,435 prescriptions with ATC codes for treatment of HCV infection from 2004 – 2022, 89,482 (99%) had a registered identity number and could be aggregated into individual treatment periods. Any treatment periods with ribavirin alone were dropped, as this has limited efficacy against HCV infection and was not part of Norwegian clinical guidelines [3, 9]. Among the 953 prescriptions without a registered identify number, 592 (62%) were dispensed between 2004 – 2010, 258 (27%) between 2011 – 2014 and 103 (11%) between 2015 – 2022.

The number of treatment periods by year and class of medicine over time are presented in Figure 1. From 2004 – 2022, there were 17,581 treatment periods among 14,313 individuals. Among those 14,313, 11,345 (80%) had received at least one course with an interferon-free DAA (J05AP07 – J05AP57). From 2015 the number of treatment periods initiated per year increased to a peak of 3,109 in 2018 (18% of all treatment periods). Subsequently, the number of treatment periods steadily decreased to 771 in 2022, the last year reported on.

Of a total 4,565 non-DAA treatment periods, 4,365 (96%) included ribavirin in combination with a pegylated interferon (L03AB10 or -11), 192 (4.2%) included a pegylated interferon only and 8 (0.2%) included a none-pegylated interferon (L03AB04 or -05) only or with ribavirin.

In 2014 (774 treatment periods), the most common course was ribavirin and interferon (n=210, 27%), followed by sofosbuvir (J05AP08) with ribavirin and interferon (n=168, 22%) and sofosbuvir with ribavirin (n=156, 20%). In 2015 (1,007 treatment periods), the most common course was sofosbuvir/ledipasvir (J05AP51, n=352, 35%), followed by sofosbuvir/ledipasvir with ribavirin (n=193, 19%) and ribavirin and interferon (n=123, 12%). In 2016 (1,135 treatment periods), the most common course included ombitasvir/paritaprevir/ritonavir (J05AP53, n=314, 28%), followed by

sofosbuvir/ledipasvir alone (n=196, 17%) and sofosbuvir, daclastavir (J05AP07) and ribavirin (n=120, 11%). In 2017 (1,887 treatment periods), the most common course was elbasvir/grazoprevir (J05AP54, n=1,092, 58%), followed by sofosbuvir/velpatasvir (J05AP55, n=397, 21%) and sofosbuvir/velpatasvir with ribavirin (n=102, 5.4%).

Since 2018, fixed dose combinations-based courses have been (almost) exclusively prescribed (n=8,192 (99%) with a fixed dose combination-based course; n=2 (0.02%) with a polymerase inhibitor-based course; n=4 (0.05%) with ribavirin/interferon only). From 2018 – 2020, sofosbuvir/velpatasvir was the most common fixed dose combination prescribed (2018: n=1,987, 64%; 2019: n=1,181, 58%; 2020: n=781, 59%), before decreasing to 14% (n=108) in 2022. Prescription of glecaprevir/pibrentasvir (J05AP57) increased from 2.2% (n=29) in 2020 to 52% (n=401) in 2022. Sofosbuvir/ledipasvir was prescribed in 30% (n=624) and 32% (n=428) of treatment periods in 2019 and 2020 respectively. Sofosbuvir/ledipasvir was rarely prescribed with ribavirin (n=18/1,333 treatment periods from 2018 – 2022). Elbasvir/grazoprevir was prescribed in 29% (n=903) and 28% (n=217) of treatment periods in 2018 and 2022, respectively. This pattern reflects national treatment guidelines, where elbasvir/grazoprevir was recommended for genotype 1 patients without cirrhosis in 2017 and 2022 [6, 7] and sofosbuvir/ledipasvir was recommended in 2019 and 2022 [7, 8].

Treatment courses by cases notified to MSIS

Of those notified cases that were treated, the number who had been dispensed at least one interferon-free direct-acting antiviral (ATC codes J05AP07, -08, -09, -51, -53, -54, -55, -56 and -57) was: All cases: 10,338/13,186 (78%); Test method RNA or antigen detection: 9,271/11,285 (82%); Still resident at the end of 2022: 9,385/11,767 (80%); Test method RNA or antigen detection and still resident at the end of 2022: 8,401/10,090 (83%). Equivalent figures also including those who had received a protease inhibitor (J05AP02, -03, -05) were 10,670/13,186 (81%), 9,553/11,285 (85%), 9,659/11,767 (82%) and 8,639/10,090 (86%).

Proportion of treatment periods that were the first for an individual

In each year, most treatment periods were first treatment periods for an individual (Figure 2). From 2012 – 2015 there was a lower proportion of first treatment periods (55% – 67%), when many individuals previously treated with ribavirin/interferon were retreated with a DAA. This increased to 89% in 2018 and 2019, when unrestricted treatment became available for the first time. In 2020, 2021 and 2022 83%, 79% and 77% of treatment periods, respectively, were the individuals first treatment period. Among the 598/3,038 (20%) treatment periods from 2020 – 2022 that were not first treatment periods, 98 (16%) received a fixed dose combination, after having been treated with ribavirin/interferon in their previous treatment period. Using the definition for complete treatment dispensation in the main manuscript, 147 (25%) had received a DAA in their previous treatment period and not been dispensed a complete course, whereas 352 (59%) had been dispensed a complete course. Among those 352 who had been dispensed a complete course in their previous period, the exact same course was dispensed again for 75 (21%), with a median time between treatment periods of 17 months (interquartile range (IQR): 9 – 31).

Treatment periods and whether individuals were notified to MSIS

Overall, 16,284 treatment periods (93%) were among individuals who had been notified to MSIS. The proportion was ≥ 90% in most years, except for 2015 (87%). From 2018 – 2022, the proportion ranged from 91% – 93% (Figure 3).

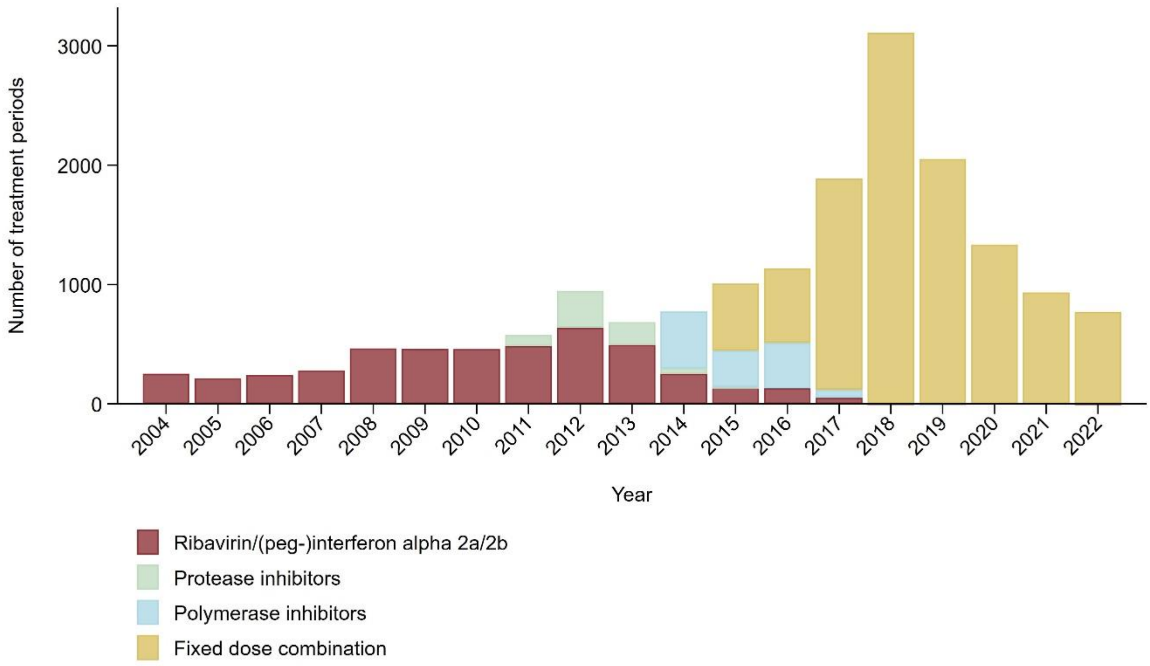

Figure 1. Number of treatment periods for hepatitis C virus infection, by year and class of medicine, Norway, 2004 – 2022.

The definition of treatment periods is presented in the text above. Ribavirin/interferon-α 2a/2b: Anatomical Therapeutic Chemical (ATC) code J05AP01 and L03AB04, -05, -10 or -11. Treatment periods with ribavirin only are excluded. Protease inhibitors: ATC codes J05AP02, J05AP03 or J05AP05. Polymerase inhibitors: ATC codes J05AP07, J05AP08 or J05AP09. Fixed dose combinations: ATC codes J05AP51, J05AP53, J05AP54, J05AP55, J05AP56 or J05AP57. For treatment periods where > 1 class of medicine was dispensed, these are presented as a) a protease inhibitor, if a protease inhibitor was dispensed and a polymerase inhibitor or fixed dose combination were not dispensed, b) a polymerase inhibitor if a polymerase inhibitor was dispensed and a fixed dose combination was not dispensed, or c) a fixed dose combination if a fixed dose combination was dispensed.

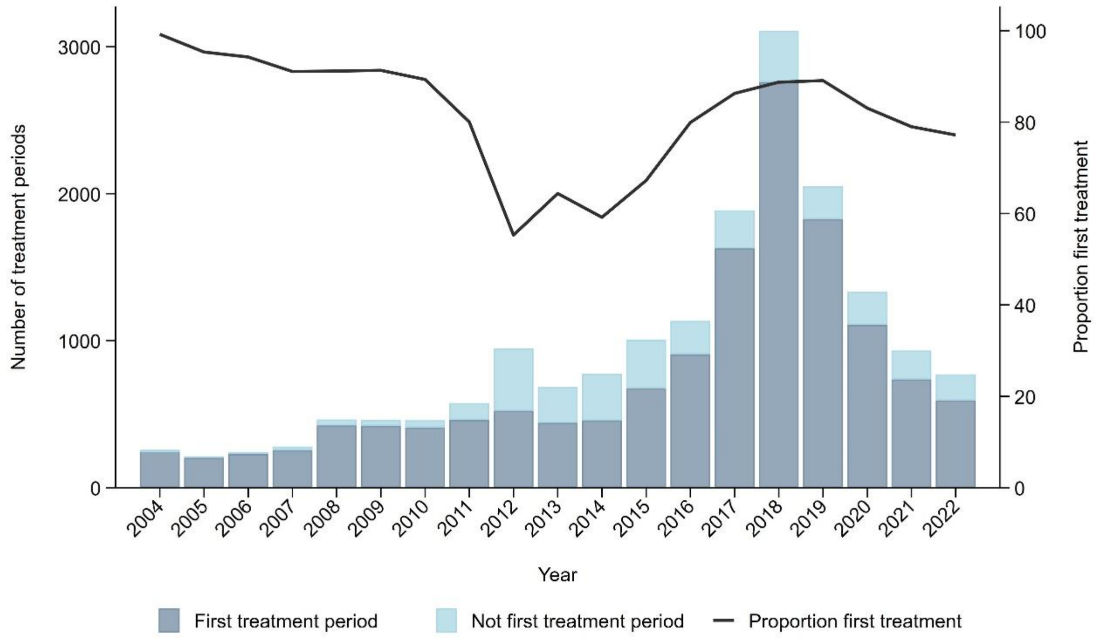

Figure 2. Number of treatment periods for hepatitis C virus infection by year and whether the treatment period was the first for an individual, Norway, 2004 – 2022.

Treatment for hepatitis C virus infection includes Anatomical Therapeutic Chemical codes J05AP and/or L03AB04, -05, -10 and -11. The definition of treatment periods is presented in the text above.

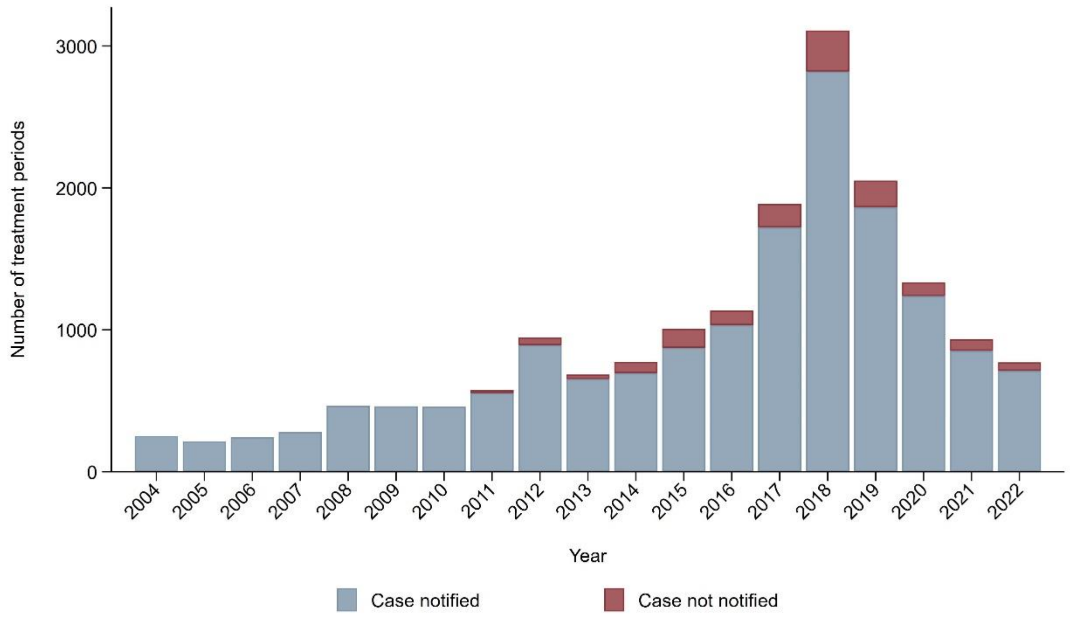

Figure 3. Number of treatment periods for hepatitis C virus infection by year and whether the individual was notified to the Norwegian Surveillance System for Communicable Diseases as a case of hepatitis C, Norway, 2004 – 2022.

Treatment for hepatitis C virus infection includes Anatomical Therapeutic Chemical codes J05AP and/or L03AB04, -05, -10 and -11. The definition of treatment periods is presented in the text above.

2. Results from alternative definition of complete DAA treatment course dispensed

In the manuscript, for each treatment period that started by November 2022 (a minimum three months of follow-up) with the prescription of at least one DAA, we determined that a complete course had been dispensed, based on the DAA(s) and number of Defined Daily Doses (DDD) prescribed in the period (defined in Table 1 in the manuscript), according to Norwegian clinical guidelines [3-8].

For some treatments the course length could vary for example by genotype and stage of liver disease [3-8], but data were lacking on these parameters. Specifically, two treatment courses could have been longer in certain patient groups. For sofosbuvir with ribavirin, according to 2014 guidelines [4] a 24-week course (168 DDD) was recommended for select patient groups in whom interferon was contraindicated e.g. liver transplant patients, some kidney transplant patients and persons with psychiatric illnesses. For sofosbuvir/ledipasvir alone, a 12-week course (84 DDD) was recommended for genotype 1 patients with cirrhosis in 2015 and 2019 guidelines [5, 8] (elbasvir/grazoprevir recommended for these patients in 2017 [6]) and genotype 4 patients in 2015 guidelines [5] (other courses recommended for these patients from 2017 [6-8]).

When defining dispensation of a complete course of sofosbuvir + ribavirin as 168 DDD (instead of 84 in manuscript) and a complete course of sofosbuvir/ledipasvir alone as 84 DDD (instead of 56 in the manuscript), 8,739 (83%) of 10,533 cases had ever had at least one complete treatment period dispensed, while 9,209 (79%) of 11,661 treatment periods were had complete treatment dispensed. The proportion with a complete course dispensed decreased to < 80% in 2014 – 2016 and 2019 – 2021. The discrepancy in results for complete course dispensation using this alternative definition are presented in Figure 4.

The lower proportion of periods with a complete course dispensed using the alternative definition suggests that the results for complete treatment dispensation presented in the manuscript are likely overestimated in 2014 – 2016 and 2019 – 2021. However, the longer courses for sofosbuvir with ribavirin and sofosbuvir/ledipasvir alone were only recommended for restricted groups of patients or rare genotypes in Norway (in the case of genotype 4). Furthermore, there was minimal discrepancy in results for the manuscript and alternative definition in 2017 and 2018, when 12 weeks with elbasvir/grazoprevir was recommended for genotype 1 patients without cirrhosis, instead of the 8 weeks with sofosbuvir/ledipasvir recommended in 2015 – 2016 and 2019 – 2021. This suggests high treatment completeness in this patient group, and that an 8-week course for sofosbuvir/ledipasvir most likely reflects that complete treatment was dispensed for most patients. We therefore consider the definition of complete DAA treatment course dispensation presented in the manuscript to most closely reflect what was prescribed in Norway.

In updated national guidelines in 2022, some 24-week courses (168 DDD) were recommended for patients with decompensated cirrhosis. These included sofosbuvir/ledipasvir or sofosbuvir/velpatasvir as an alternative (i.e. not first choice) course for non-genotype 3 patients, sofosbuvir/velpatasvir as one of two possible first choice courses for genotype 3 patients and sofosbuvir/velpatasvir + ribavirin for retreatment of treatment failures [7]. These 24-week courses are not further considered in additional alternative definitions of complete treatment dispensation. Of 771 treatment periods in 2022, 37 (4.8%) received sofosbuvir/ledipasvir, 100 (13%) sofosbuvir/velpatasvir and 6 (0.8%) sofosbuvir/velpatasvir + ribavirin. Of these courses, 1, 16 and 2 respectively were dispensed > 12 weeks (84 DDD). Most patients (29, 68 and 3, respectively) were dispensed a number of DDD that reflected a complete 8- or 12-week course (56 or 84 DDD). Of the 771 treatment periods, 13 (1.7%)

were courses dedicated to retreatment of treatment failures in national guidelines (sofosbuvir/velpatasvir + ribavirin or sofosbuvir/velpatasvir/voxilaprevir (J05AP56)) [7].

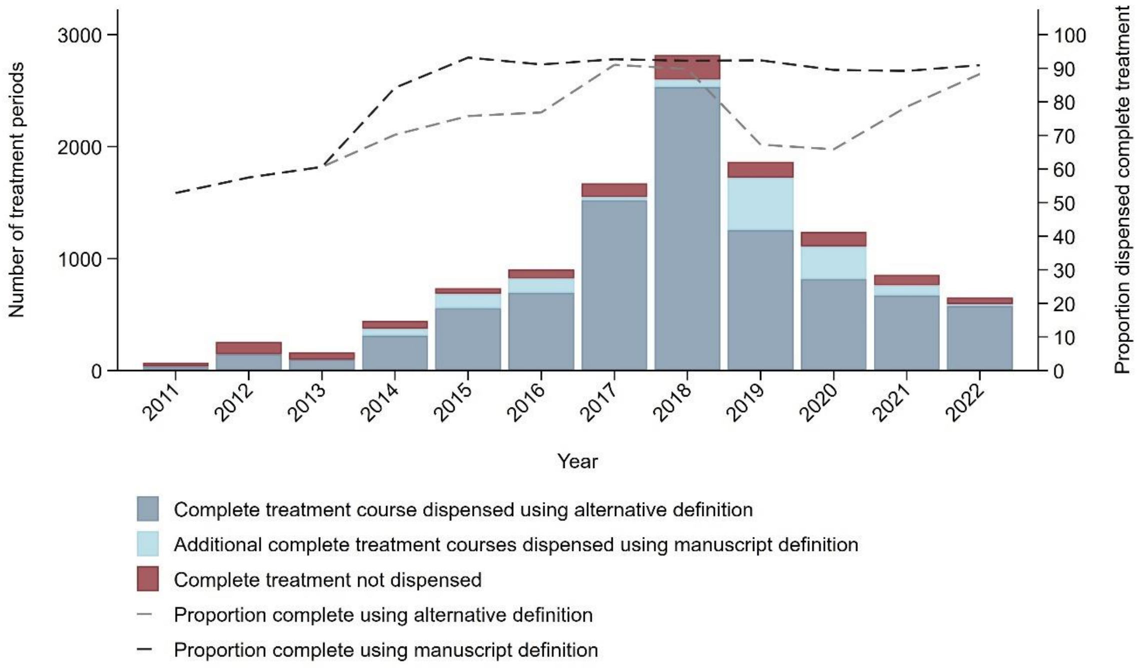

Figure 4. Number and proportion of treatment periods for hepatitis C virus infection with prescription of at least one direct-acting antiviral, by definition of complete DAA treatment course dispensation and year, notified cases of hepatitis C virus infection, Norway, January 2011 – November 2022.

'Treatment periods for hepatitis C virus (HCV) infection with prescription of at least one direct-acting antiviral' defined as being dispensed a prescription for Anatomical Therapeutic Chemical codes J05AP02 – J05AP57. The figure includes 11,661 different treatment periods for 10,533 cases. Persons infected with HCV may undergo several treatment periods, either due to treatment incompletion, failure or reinfection. As the data we had access to from the Norwegian Prescribed Drug Registry were until the end of February 2023, the year 2022 excludes treatment periods where the first prescription was in December 2022, to ensure a minimum three-months follow-up for all courses. Being dispensed a complete course defined as described in Table 1 in the main manuscript. For the alternative definition, a complete course of sofosbuvir (J05AP08) with ribavirin was defined as 24 weeks (168 DDD) and sofosbuvir/ledipasvir (J05AP51) without ribavirin complete at 12 weeks (84 DDD). In the manuscript, a complete course of sofosbuvir with ribavirin was defined as 12 weeks (84 DDD) and a complete course of sofosbuvir/ledipasvir without ribavirin was defined as 8 weeks (56 DDD). Further details on the treatment courses dispensed by year are also presented above (see Figure 1).

3. Characteristics of notified cases of HCV infection who were untreated and still resident at the end of 2022

Table 1. Characteristics of notified cases of hepatitis C virus infection who were untreated and still resident at the end of 2022, by test method, Norway.

	RNA/antigen positive		Antibody positive, unknown RNA/ antigen status		All cases[a]	
Characteristic	Number	%	Number	%	Number	%
Overall	2,088	100	2,830	100	5,246	100
Year of diagnosis[b]						
1990 – 1991	19	0.9	79	2.8	381	7.3
1992 – 2007	208	10.0	483	17.1	711	13.6
2008 – 2010	614	29.4	1,125	39.8	1,755	33.5
2011 – 2013	384	18.4	745	26.3	1,138	21.7
2014 – 2015	228	10.9	398	14.1	626	11.9
2016 – 2017	198	9.5	0	0.0	198	3.8
2018 – 2019	162	7.8	0	0.0	162	3.1
2020 – 2021	126	6.0	0	0.0	126	2.4
2022	149	7.1	0	0.0	149	2.8
Age in years at the end of 2022						
0 – 2	0	0.0	0	0.0	0	0.0
3 – 14	15	0.7	5	0.2	20	0.4
15 – 24	12	0.6	12	0.4	24	0.5
25 – 34	290	13.9	151	5.3	447	8.5
35 – 44	561	26.9	666	23.5	1,239	23.6
45 – 54	584	28.0	977	34.5	1,611	30.7
55 – 64	424	20.3	739	26.1	1,314	25.0
≥ 65	202	9.7	280	9.9	591	11.3
Sex						
Female	843	40.4	1262	44.6	2,233	42.6
Male	1,245	59.6	1568	55.4	3,013	57.4
County of residence at the end of 2022[c]						
Agder	218	10.4	140	4.9	373	7.1
Innlandet	139	6.7	167	5.9	344	6.6
Møre og Romsdal	77	3.7	62	2.2	149	2.8
Nordland	83	4.0	82	2.9	174	3.3
Oslo	244	11.7	519	18.3	837	16.0
Rogaland	281	13.5	306	10.8	609	11.6
Troms og Finnmark	93	4.5	77	2.7	180	3.4
Trøndelag	128	6.1	131	4.6	284	5.4
Vestfold og Telemark	224	10.7	311	11.0	566	10.8
Vestland	128	6.1	297	10.5	450	8.6
Viken	450	21.6	728	25.7	1,247	23.8
Unknown	23	1.1	10	0.4	33	0.6

Table 1 cont.

	RNA/antigen positive		Antibody positive, unknown RNA/ antigen status		All cases[a]	
Country of birth[d]						
Africa	28	1.3	34	1.2	62	1.2
Asia[e]	53	2.5	53	1.8	107	2.0
Europe	49	2.3	85	3.0	136	2.6
Iran	7	0.3	12	0.4	19	0.4
Latvia	11	0.5	4	0.1	15	0.3
Lithuania	20	1.0	13	0.5	33	0.6
North America	4	0.2	13	0.5	17	0.3
Norway	1,742	83.4	2,313	81.7	4,088	77.9
Pakistan	16	0.8	58	2.1	75	1.4
Poland	20	1.0	35	1.2	55	1.1
Russia	25	1.2	34	1.2	59	1.1
Somalia	11	0.5	17	0.6	31	0.6
Sweden	15	0.7	25	0.9	40	0.8
Central/South America	12	0.6	15	0.5	28	0.5
Ukraine	39	1.9	5	0.2	44	0.8
Vietnam	10	0.5	15	0.5	26	0.5
'Overseas'	8	0.4	34	1.2	43	0.8
Unknown[f]	18	0.9	65	2.3	368	7.0
Reported route of transmission						
Injecting drugs	1,186	56.8	1,361	48.1	2,570	49.0
Blood contact[g]	49	2.3	68	2.4	118	2.3
Heterosexual contact	10	0.5	3	0.1	13	0.3
Homosexual contact	8	0.4	2	0.1	10	0.2
Sexual contact, unspecified	38	1.8	61	2.2	103	2.0
Perinatal	16	0.8	6	0.2	22	0.4
'Other'	3	0.1	6	0.2	9	0.2
Unknown	778	37.3	1,323	46.8	2,401	45.8
Reported place of infection						
Norway	1,607	77.0	2,385	84.3	4,024	76.7
Overseas	189	9.1	223	7.9	427	8.1
Unknown	292	14.0	222	7.8	795	15.2
Test method						
Antibody detection	–	–	–	–	2,830	53.9
RNA/antigen detection	–	–	–	–	2,088	39.8
Unknown[f]	–	–	–	–	328	6.3

Table 1 cont.

[a] Includes 328 cases with unknown test method.

[b] Aggregated into periods based on the timing of changes in the national case definition for cases of HCV infection, notable changes in available treatment or treatment guidelines and the COVID-19 pandemic. From 1990 – 1991 and 2008 – 2015, all anti-HCV antibody, HCV RNA and HCV antigen positive cases were notifiable. From 1992 – 2007, only acute cases were notifiable. Chronic cases diagnosed in this period were notified from 2008 onwards. Since 2016, only RNA and core antigen positive cases have been notifiable. From 1990 – 2010, only ribavirin and/or interferon-α were prescribed as treatment for HCV infection. Protease inhibitors (J05AP02, -03 and -05) were introduced in 2011. Interferon-free polymerase inhibitors (J05AP07, -08 and -09) were introduced in 2014, and fixed dose combinations (J05AP51, -53, -54, -55, -56 and -57) from 2015 onwards. All restrictions on treatment access in Norway were removed in February 2018. The acute phase of the COVID-19 pandemic was from 2020 until early 2022. Follow-up for cases diagnosed in 2022 is incomplete (includes treatment received up to the end of February 2023).

[c] Based on county classification until the end of 2023.

[d] Countries with ≥ 100 cases reported in the entire study period presented individually. All other countries are grouped by global region.

[e] Includes two cases where the place of birth was reported as 'Oceania'.

[f] Almost exclusively reported in 1990 – 1991 (for all cases, country of birth: n=273, 74%; test method: n=283, 86%).

[g] Such as a needle-stick injury or blood transfusion.

Treatment for hepatitis C virus (HCV) infection defined as Anatomical Therapeutic Chemical codes J05AP and/or L03AB04, -05, -10 and -11. Those who had a positive test for HCV RNA or core antigen after their last prescription were analysed as untreated cases (48 among those still resident at the end of 2022). Those who had received ribavirin alone are also considered untreated cases, as this was ineffective against HCV infection and not part of Norwegian clinical guidelines [3, 9].

4. Treatment uptake by county of residence and country of birth

Table 2. County of residence and country of birth of notified cases of hepatitis C virus infection and those with treatment dispensed, according to test method and residence status, Norway, 1990–2022.

Characteristic	All cases			Test method RNA or antigen detection			Still resident at the end of 2022			Test method RNA or antigen detection and still resident at the end of 2022		
	Number of cases	Dispensed treatment		Number of cases	Dispensed treatment		Number of cases	Dispensed treatment		Number of cases	Dispensed treatment	
		Number	%		Number	%		Number	%		Number	%
Overall	22,048	13,186	60	15,100	11,285	75	17,013	11,767	69	12,178	10,090	83
County of residence at diagnosis[a]												
Agder	1,411	882	63	1,127	814	72	1,136	795	70	938	738	79
Innlandet	1,331	827	62	1,004	771	77	1,039	744	72	830	697	84
Møre og Romsdal	618	389	63	462	345	75	510	348	68	388	307	79
Nordland	813	526	65	602	459	76	631	456	72	487	407	84
Oslo	4,269	2,266	53	2,465	1,855	75	2,963	1,966	66	1,866	1,603	86
Rogaland	2,343	1,309	56	1,610	1,107	69	1,825	1,182	65	1,295	1,004	78
Troms og Finnmark	876	606	69	756	582	77	736	550	75	636	527	83
Trøndelag	1,340	848	63	1,033	781	76	1,053	766	73	848	711	84
Vestfold og Telemark	2,461	1,531	62	1,825	1,381	76	1,946	1,384	71	1,483	1,248	84
Vestland	1,920	1,183	62	921	711	77	1,534	1,076	70	774	645	83
Viken	4,612	2,792	61	3,243	2,452	76	3,626	2,491	69	2,621	2,194	84
Unknown	54	27	50	52	27	52	14	9	64	12	9	75
Country of birth[b]												
Africa	265	163	62	187	140	75	207	145	70	153	125	82
Asia[c]	432	291	67	329	256	78	372	265	71	287	234	82
Europe	676	435	64	492	379	77	521	385	74	386	337	87
Iran	106	71	67	73	59	81	83	64	77	60	53	88
Latvia	104	79	76	89	72	81	83	68	82	74	63	85
Lithuania	218	157	72	185	146	79	166	133	80	142	122	86
North America	80	54	68	54	46	85	60	43	72	41	37	90
Norway	16,839	10,450	62	12,165	9,036	74	13,489	9,401	70	9,881	8,139	82

Pakistan	404	277	69	266	219	82	314	239	76	203	187	92
Poland	279	191	68	203	163	80	221	166	75	161	141	88
Russia	259	177	68	189	149	79	221	162	73	162	137	85
Somalia	120	69	58	85	64	75	87	56	64	62	51	82
Sweden	283	175	62	223	164	74	183	143	78	150	135	90
Central/South America	126	84	67	92	70	76	100	72	72	73	61	84
Ukraine[d]	177	123	69	168	120	71	151	107	71	143	104	73
Vietnam	143	98	49	106	85	80	108	82	76	81	71	88
'Overseas'	183	90	69	102	60	59	115	72	63	56	48	86
Unknown[e]	1,354	202	15	92	57	62	532	164	31	63	45	71

[a] Based on county classification until the end of 2023. The current county of residence among those still resident but who had not received treatment is presented in part 4. In the manuscript Mid Norway = counties Møre and Romsdal, and Trøndelag. North Norway = counties Nordland, and Troms and Finnmark. South-East Norway (excluding Oslo) = counties Agder, Innlandet, Vestfold and Telemark, and Viken. West Norway = counties Rogaland and Vestland.

[b] Countries with ≥ 100 cases reported in the entire study period presented individually. All other countries are grouped by global region.

[c] Includes four cases where the place of birth was reported as 'Oceania'. Oceania is not presented as its own category, due to low numbers.

[d] Among the 143 cases born in Ukraine who were diagnosed by RNA or antigen detection and were still resident at the end of 2022, 100 (70%) were diagnosed in 2022. Supposed lower treatment uptake in this group is therefore likely due to data availability at the time of extraction.

[e] Almost exclusively reported in 1990 – 1991 (for all cases, country of birth: n=1,068, 79%; test method: n=1,110, 88%).

5. Year of treatment by year of diagnosis

Among the 13,098 cases treated from 2004 – 2022, 12,769 (97%) started a treatment period in the same month as or following a positive test or were undergoing a treatment period at the date of positive test. The remaining 329 (2.5%) cases had finished their most recent treatment period when diagnosed. Among these 329, the reported test method was anti-HCV antibody for 327 and unknown for two.

Table 3. Year of diagnosis and year of first treatment period for hepatitis C virus infection that followed or overlapped with the year and month of diagnosis, notified cases of hepatitis C virus infection, Norway, 1990 – 2022.

Year of treatment	Year of diagnosis																		
	1990 – 1991		1992 – 2007		2008 – 2010		2011 – 2013		2014 – 2015		2016 – 2017		2018 – 2019		2020 – 2021		2022		
	Nr.	%	Nr.	%	Nr.	%	Nr.	%	Nr.	%	Nr.	%	Nr.	%	Nr.	%	Nr.	%	Total
2004	14	16.1	72	82.8	1	1.1	0	0.0	0	0.0	0	0.0	0	0.0	0	0.0	0	0.0	87
2005	11	20.8	42	79.2	0	0.0	0	0.0	0	0.0	0	0.0	0	0.0	0	0.0	0	0.0	53
2006	16	23.2	52	75.4	1	1.4	0	0.0	0	0.0	0	0.0	0	0.0	0	0.0	0	0.0	69
2007	18	15.5	70	60.3	28	24.1	0	0.0	0	0.0	0	0.0	0	0.0	0	0.0	0	0.0	116
2008	20	5.0	95	23.8	283	70.9	0	0.0	0	0.0	0	0.0	0	0.0	0	0.0	0	0.0	399
2009	10	2.4	91	21.8	315	75.5	1	0.2	0	0.0	0	0.0	0	0.0	0	0.0	0	0.0	417
2010	14	3.4	63	15.3	335	81.3	0	0.0	0	0.0	0	0.0	0	0.0	0	0.0	0	0.0	412
2011	12	2.7	58	13.0	298	67.0	77	17.3	0	0.0	0	0.0	0	0.0	0	0.0	0	0.0	445
2012	13	2.6	63	12.4	212	41.8	218	42.9	0	0.0	0	0.0	0	0.0	0	0.0	0	0.0	507
2013	2	0.5	45	10.6	143	33.7	234	55.2	0	0.0	0	0.0	0	0.0	0	0.0	0	0.0	424
2014	9	2.2	38	9.4	121	30.0	187	46.3	49	12.1	0	0.0	0	0.0	0	0.0	0	0.0	404
2015	32	5.4	60	10.2	163	27.6	163	27.6	172	29.2	0	0.0	0	0.0	0	0.0	0	0.0	590
2016	37	4.4	76	9.0	207	24.6	164	19.5	229	27.2	128	15.2	0	0.0	0	0.0	0	0.0	841
2017	54	3.6	161	10.6	374	24.7	306	20.2	243	16.0	376	24.8	2	0.1	0	0.0	0	0.0	1,516
2018	74	2.9	208	8.3	582	23.1	446	17.7	321	12.7	568	22.5	321	12.7	0	0.0	0	0.0	2,520
2019	49	2.9	138	8.2	367	21.8	239	14.2	171	10.2	148	8.8	569	33.8	0	0.0	0	0.0	1,681
2020	19	1.8	90	8.6	204	19.6	146	14.0	83	8.0	68	6.5	202	19.4	231	22.1	0	0.0	1,043
2021	12	1.8	41	6.0	107	15.7	87	12.8	41	6.0	33	4.9	29	4.3	330	48.5	0	0.0	680
2022	9	1.6	21	3.7	63	11.1	54	9.5	28	4.9	25	4.4	27	4.8	104	18.4	235	41.5	566

6. Data behind figure 1

In Figure 1 in the manuscript, the year of treatment is the year of the first treatment period (defined in part 1 of this supplement) that occurred in the month of diagnosis or later. The 332 cases who had finished their most recent treatment period when diagnosed (330 anti-HCV antibody positive, 2 with unknown test method) are considered as treated when diagnosed. The figure for 'All cases' includes 1,254 cases with unknown test method. The figure excludes 65 cases (53 RNA or antigen positive, 11 antibody positive, 1 with unknown test method) who could not be linked to a registration in the National Population Register, and thus had unknown data on residence status. Also, 86 cases in the dataset (81 RNA or antigen positive, 5 antibody positive) known to have been treated in January or February 2023 are considered as untreated in the figure.

Table 4. Data behind Figure 1 in the manuscript.

Year	Test method	Treated	Not treated	Proportion treated (%)
2004	All cases	151	2929	5
2005	All cases	270	3075	8
2006	All cases	412	3249	11
2007	All cases	592	3469	15
2008	All cases	1007	6046	14
2009	All cases	1419	7550	16
2010	All cases	1809	8540	17
2011	All cases	2237	9354	19
2012	All cases	2722	9983	21
2013	All cases	3110	10538	23
2014	All cases	3458	10996	24
2015	All cases	3985	11239	26
2016	All cases	4733	11040	30
2017	All cases	6153	10067	38
2018	All cases	8548	8079	51
2019	All cases	10100	6847	60
2020	All cases	10939	6105	64
2021	All cases	11410	5668	67
2022	All cases	11713	5363	69
2004	Positive RNA or antigen test at diagnosis	28	1370	2
2005	Positive RNA or antigen test at diagnosis	52	1487	3
2006	Positive RNA or antigen test at diagnosis	85	1621	5
2007	Positive RNA or antigen test at diagnosis	141	1800	7
2008	Positive RNA or antigen test at diagnosis	467	3583	12
2009	Positive RNA or antigen test at diagnosis	834	4538	16
2010	Positive RNA or antigen test at diagnosis	1155	5201	18
2011	Positive RNA or antigen test at diagnosis	1526	5708	21
2012	Positive RNA or antigen test at diagnosis	1945	6020	24
2013	Positive RNA or antigen test at diagnosis	2282	6293	27
2014	Positive RNA or antigen test at diagnosis	2600	6551	28
2015	Positive RNA or antigen test at diagnosis	3061	6635	32
2016	Positive RNA or antigen test at diagnosis	3738	6603	36
2017	Positive RNA or antigen test at diagnosis	4984	5914	46
2018	Positive RNA or antigen test at diagnosis	7111	4292	62
2019	Positive RNA or antigen test at diagnosis	8515	3309	72
2020	Positive RNA or antigen test at diagnosis	9288	2729	77

2021	Positive RNA or antigen test at diagnosis	9736	2384	80
2022	Positive RNA or antigen test at diagnosis	10038	2191	82
2004	Positive antibody test at diagnosis	116	915	11
2005	Positive antibody test at diagnosis	204	962	17
2006	Positive antibody test at diagnosis	307	1016	23
2007	Positive antibody test at diagnosis	421	1080	28
2008	Positive antibody test at diagnosis	503	1877	21
2009	Positive antibody test at diagnosis	545	2442	18
2010	Positive antibody test at diagnosis	609	2784	18
2011	Positive antibody test at diagnosis	660	3099	18
2012	Positive antibody test at diagnosis	723	3423	17
2013	Positive antibody test at diagnosis	775	3711	17
2014	Positive antibody test at diagnosis	804	3927	17
2015	Positive antibody test at diagnosis	861	4112	17
2016	Positive antibody test at diagnosis	924	3960	19
2017	Positive antibody test at diagnosis	1081	3705	23
2018	Positive antibody test at diagnosis	1316	3387	28
2019	Positive antibody test at diagnosis	1445	3168	31
2020	Positive antibody test at diagnosis	1507	3020	33
2021	Positive antibody test at diagnosis	1525	2939	34
2022	Positive antibody test at diagnosis	1529	2843	35

7. Data behind figure 2

Table 5. Data behind Figure 2 in the manuscript.

Year of diagnosis	Number of cases	Number treated	Median number of months to treatment start	Lower quartile	Upper quartile
2004	237	129	134	66	169
2005	220	143	118	41	157
2006	267	155	83	27	141
2007	344	193	71	13	130
2008	3186	1890	69	11	119
2009	2141	1175	81	17	110
2010	1609	925	72	15	98
2011	1499	906	68	15	87
2012	1389	800	52	14	76
2013	1233	731	47	17	63
2014	1132	678	38	16	52
2015	1128	703	29	11	42
2016	894	701	17	7	28
2017	766	646	8	5	15
2018	745	631	5	3	9
2019	631	522	4	2	8
2020	467	375	3	1	7
2021	371	292	3	2	6
2022	457	288	2	1	4

8. References

1 Helsedata. Data sources. Available at: https://helsedata.no/en/data-sources/?page=1&sort=0. Accessed 15 March 2024.

2 WHO Collaborating Centre for Drug Statistics Methodology. ATC/DDD Index 2023. 2023. Available at: https://www.whocc.no/atc_ddd_index/. Accessed 15 March 2024.

3 Dalgard O, Konopski Z, Bosse FJ, Nordstrand B, Sandvei P, Karlsen L, et al. Hepatitt C – utredning og behandling [In Norwegian]. Tidsskr Nor Legeforen. 2011. http://dx.doi.org/10.4045/tidsskr.10.02401.

4 Norwegian Medical Association. Faglig veileder for utredning og behandling av hepatitt C [In Norwegian]. 2014. Available at: https://gastroenterologen.no/2014/10/faglig-veileder-for-utredning-og-behandling-av-hepatitt-c. Accessed 15 March 2024.

5 Norwegian Medical Association. Faglig veileder for utredning og behandling av hepatitt C [In Norwegian]. 2015. Available at: https://gastroenterologen.no/filer/Veileder-Revisjon-mars-2015.pdf. Accessed 15 March 2024.

6 Norwegian Medical Association. Faglig veileder for utredning og behandling av hepatitt C hos voksne [In Norwegian]. 2017. Available at: https://www.legeforeningen.no/contentassets/69820f918014482a8003801d26adced3/hcv-v7-2017.pdf. Accessed 15 March 2024.

7 Norwegian Medical Association. Faglig veileder for utredning og behandling av hepatitt C hos voksne [In Norwegian]. 2019. Available at: https://www.legeforeningen.no/contentassets/be28359cd11745a8bbc090e93d9b9c90/veileder-revisjon-hcv.pdf. Accessed 15 March 2024.

8 Norwegian Medical Association. Faglig veileder for utredning og behandling av hepatitt C [In Norwegian]. 2022. Available at: https://www.legeforeningen.no/contentassets/7e304300c0b5464eaf682fa7b70dd659/veileder-hepatitt-c-2022.pdf. Accessed 15 March 2024.

9 Manns MP, Maasoumy B. Breakthroughs in hepatitis C research: from discovery to cure. Nat Rev Gastroenterol Hepatol. 2022;19(8):533-50.
